# Patient-reported outcomes after immediate and delayed DIEP-flap breast reconstruction in the setting of post-mastectomy radiation therapy—results of the multicenter UMBRELLA breast cancer cohort

**DOI:** 10.1007/s10549-025-07613-w

**Published:** 2025-02-03

**Authors:** Britt A. M. Jansen, Claudia A. Bargon, Maria A. Bouman, Dieuwke R. Mink van der Molen, Emily. L. Postma, Femke van der Leij, Erik Zonnevylle, Quinten Ruhe, Sven E. Bruekers, Wiesje Maarse, Sabine Siesling, Danny A. Young-Afat, Annemiek Doeksen, Helena M. Verkooijen, UMBRELLA study group

**Affiliations:** 1 Division of Imaging and Oncology, University Medical Centre Utrecht, Cancer Centre, Utrecht, The Netherlands; 2 Department of Surgery, St. Antonius Hospital, Soestwetering 1, 3543 AZ Utrecht, The Netherlands; 3 Department of Plastic and Reconstructive Surgery, St. Antonius Hospital, Utrecht, The Netherlands; 4 Department of Radiation Oncology, University Medical Centre Utrecht, Cancer Centre, Utrecht, The Netherlands; 5 Department of Plastic Surgery, Meander Medical Center, Amersfoort, The Netherlands; 6 Department of Plastic Surgery, Diakonessenhuis, Zeist, The Netherlands; 7 Department of Plastic, Reconstructive and Hand Surgery, Amsterdam University Medical Centre, Amsterdam, The Netherlands; 8 Department of Research, Netherlands Comprehensive Cancer Organisation (IKNL), Utrecht, The Netherlands; 9 Department of Health Technology and Services Research Technical Medical Centre, University of Twente, Enschede, The Netherlands; 10 Utrecht University, Utrecht, The Netherlands

**Keywords:** Breast cancer, Oncoplastic breast surgery, DIEP-flap breast reconstruction, Patient-reported outcome, Late radiation toxicity

## Abstract

**Purpose:**

Timing of Deep Inferior Epigastric artery Perforator (DIEP)-flap breast reconstruction in the context of post-mastectomy radiotherapy for breast cancer patients is topic of debate. We compared the impact of immediate (before radiotherapy) versus delayed (after radiotherapy) DIEP-flap breast reconstruction (IBR versus DBR) on short- and long-term patient-reported outcomes (PROs).

**Methods:**

Within the prospective, multicenter breast cancer cohort (UMBRELLA), we identified 88 women who underwent immediate or delayed DIEP-flap breast reconstruction and received PMRT. At 6 and 12 months post-mastectomy, as well as on long-term (≥ 12 months post-reconstruction) body image, breast symptoms, physical functioning, and pain were measured by EORTC-QLQ-30/BR23. Additionally, long-term evaluation included satisfaction with breast(s), physical well-being and self-reported adverse effects of radiation as measured by BREAST-Q, and late treatment toxicity. PROs were compared between groups using independent sample *T*-test.

**Results:**

IBR was performed in 56 patients (64%) and DBR in 32 patients (36%), with 15 months of median time to reconstruction. At 6 and 12 months post-mastectomy, better body image and physical functioning were observed after IBR. No statistically nor clinically relevant differences were observed in long-term EORTC and BREAST-Q outcomes (median follow-up 37–41 months for IBR vs. 42–46 months for DBR). Patients with IBR reported more fibrosis and movement restriction (median follow-up 29 vs. 61 months, resp.).

**Conclusion:**

Long-term PROs were comparable for patients with IBR and DBR, despite more patient-reported fibrosis and movement restriction after IBR. Therefore, both treatment pathways can be considered when opting for autologous breast reconstruction in the setting of PMRT.

**Supplementary Information:**

The online version contains supplementary material available at 10.1007/s10549-025-07613-w.

## Introduction

In the context of breast cancer, the loss of a breast is frequently associated with traumatic emotional experiences, resulting in diminished self-esteem and body image perception. [1] For many women, the option of post-mastectomy reconstructive surgery holds significant value in the treatment of breast cancer, enhancing health-related quality of life (HR-QoL) and satisfaction. [2] The use of autologous tissue offers multiple advantages compared to implant-based techniques, particularly in the setting of post-mastectomy radiation therapy (PMRT). [3] The deep inferior epigastric artery perforator (DIEP) flap is the gold standard when considering an autologous BR. [4] Immediate autologous breast reconstruction (IBR) is becoming increasingly common, primarily due to its cosmetic and psychological benefits. [5–12].

Post-mastectomy radiotherapy (PMRT) has significant impact on locoregional disease control and the reduction of disease-related mortality, particularly in high-risk subgroups (i.e., locally advanced breast cancer, multiple positive lymph nodes, and positive surgical margins). [13] However, PMRT can negatively influence the options for, and outcomes after breast reconstruction. [3, 6] Preoperative indications for PMRT are not always definitive, as indications for PMRT primarily depend on pathological staging following oncological resection. Moreover, recent changes to the National Comprehensive Cancer Network (NCCN) guidelines have expanded the indication for PMRT by recommending PMRT in patients with one to three positive lymph nodes. [14] As a result more patients are offered PMRT, increasingly posing a challenge for clinical decision-making.

A global debate persists regarding the optimal timing for autologous breast reconstruction in the context of PMRT, specifically whether to perform the reconstruction prior to PMRT or to postpone it until after completion of PMRT. High complication and revision rates for irradiated autologous flap reconstructions have been described, including scarring (fibrosis), fat necrosis, and atrophy. [15–17] However, more recent data, informed by modern-day radiotherapy and reconstructive practices, suggest that the complication rates and patient-reported outcomes (PROs) associated with IBR followed by PMRT are comparable to those with DBR subsequent to PMRT [18–21].

Due to the global controversy and the lack of high-quality scientific evidence, the timing of breast reconstruction in the setting of PMRT exhibits considerable variability and is still mainly based on clinicians’ preferences and experience. [22, 23] This variability in clinical practice provided a unique opportunity to compare PROs between the two different treatment pathways [24].

The primary aim of this study was to assess long-term PROs in breast cancer patients who underwent mastectomy with PMRT and either immediate or delayed DIEP-flap breast reconstruction. Additionally, we aimed to compare the longitudinal PROs during the initial year after mastectomy and reconstruction between patients with IBR to those with DBR. We hypothesize that there will be no significant difference in long-term PROs, but anticipate that patients undergoing immediate reconstruction will demonstrate better short-term outcomes.

## Methods

### Study design and participants

Patients were recruited from the ‘Utrecht cohort for Multiple BREast cancer intervention studies and Long-term evaluAtion’ (UMBRELLA) cohort. [25] UMBRELLA is a prospective, multicenter cohort including breast cancer patients. UMBRELLA participants are aged ≥ 18 years, and all have histologically proven invasive or in situ breast carcinoma. Participants agreed to the longitudinal collection of clinical data and PRO questionnaires at regular time intervals, i.e., 3 and 6 months after inclusion, and every 6 months thereafter up to 10 years after inclusion. The UMBRELLA study adheres to the Dutch Law on Medical Research Involving Human Subjects (WMO) and the Declaration of Helsinki (version 2013). Approval by the Medical Research Ethics Committee (MREC) Utrecht (NL52651.041.15, MEC15/165) was obtained for all sites. The study is registered on clinicaltrials.gov (NCT02839863).

Data of patients participating in the UMBRELLA cohort are regularly linked to the Netherlands Cancer Registry (NCR) to obtain pathology, clinical, treatment, and follow-up data. Data of the NCR is gathered in all hospitals in the Netherlands by trained data managers directly from patients’ files. Linkage is approved by the Committee of Privacy (K20.108).

Patients with immediate DIEP-flap breast reconstruction received PMRT on the chest wall and neo-breast, approximately 6–8 weeks after completing mastectomy and reconstruction. Patients undergoing delayed DIEP-flap remained flat after the surgery and received PMRT 6–8 weeks after surgery on the chest wall. Patients underwent DIEP-flap reconstruction at least 6 months after having completed PMRT. The majority of patients were treated with a dose of 42.56 Gy in 16 fractions or 40.05 Gy in 15 fractions. If indicated, patients received RT on the chest wall plus regional lymph nodes (axillary (level I, II, and III), medial part of the supraclavicular fossa, and/or internal mammary).

### Data collection

From the UMBRELLA cohort, we selected all patients who underwent mastectomy and immediate or delayed DIEP-flap BR and received PMRT between October 2013 and June 2022. Exclusion criteria were radiotherapy prior to mastectomy and implant-based BR prior to DIEP-flap BR. As only three patients had undergone delayed-immediate reconstruction (i.e., with the use of a tissue expander) and did not respond to the questionnaires, these patients were excluded from analysis to establish more homogeneous groups.

Clinical data, as retrieved from the Netherlands Cancer Registry (NCR) or collected in the context of UMBRELLA included age, body mass index (BMI) at reconstruction, smoking status, pathological TNM-staging classification (AJCC 7th/8th edition), [26] axillary treatment, (neo-)adjuvant systemic therapy, radiation therapy, (date of) IBR and use of tissue expander (i.e., delayed-immediate reconstruction). Since DBR is not recorded in the NCR, patient files were reviewed to identify DBR and the date of DBR was documented.

To evaluate long-term PROs, the most recently completed European Organisation for Research and Treatment of Cancer Quality of Life Questionnaire (EORTC-QLQ), BREAST-Q and late treatment toxicity (LTT) questionnaires were selected for each patient, with a minimum of 12 months post-reconstruction. The EORTC-QLQ includes a core questionnaire assessing QoL of cancer patients (QLQ-C30) and a breast cancer specific module (QLQ-BR23). Domains of interest included body image, breast symptoms, physical functioning, and pain. [27] Items are rated on a 4-point Likert scale ranging from 1 ‘not et al’ to ‘very much’. For each subscale, a summary score on a scale from 0–100 was calculated according to the EORTC manual. [28] Higher scores represent better outcomes for body image and physical functioning, whereas lower scores for pain and breast symptoms represent better outcomes. The BREAST-Q measure is a validated tool to assess patient satisfaction and HR-QoL and has become the gold standard to assess PROs. [29] The BREAST-Q Reconstruction Module has been applied within our cohort since August 2018. Subscales relevant for this study included satisfaction with breast(s), physical well-being, and adverse effects of radiation. Each subscale consists of multiple items. Each item is converted into scores ranging from 0 to 100. Higher scores indicate better outcomes. Mean scores were calculated and summarized for both groups according the BREAST-Q manual. Self-reported late treatment toxicity (LTT) was measured through the LTT questionnaire (Appendix I, Supplementary Information). [30] This questionnaire includes 13 questions based on Common Terminology Criteria for Adverse Events (CTCAE v4.0) and EORTC-QLQ-C30/BR23 subdomains and evaluates breast and/or chest wall pain, firmness of the breast, lymphedema, and movement restriction. All questions were scored on a 4-point Likert scale from ‘not at all’ to ‘very much’. Missing data were addressed according to the scoring manual.

To evaluate the trajectory of PROs in the initial year following mastectomy and reconstruction, outcomes of the before-mentioned EORTC-QLQ-C30/BR23 domains were measured approximately at 6 and 12 months post-mastectomy, and approximately at 6 and 12 months post-reconstruction (with a window of 3 months). For patients with IBR, post-mastectomy PROs align inherently with the post-reconstruction PROs, while post-mastectomy PROs for patients with DBR provide insight into their transitional ‘flat state’ phase. Patients who had their reconstruction within 12 months (*n* = 6) were excluded from post-mastectomy comparison.

Minimal clinically important differences (MCIDs) are used in PROs to identify the smallest meaningful change perceived as clinically relevant. [31] By using MCIDs, healthcare providers can better understand patients’ perspectives. MCID values for most domains of the EROTC-QLQ-C30/BR23 and BREAST-Q have been established for breast cancer patients. For the EORTC-QLQ-C30/BR23, MCIDs of 5 for physical functioning and 6 for pain were determined. For body image and breast symptoms, no official MIDs have been developed yet. For the BREAST-Q, MIDs of 4 for satisfaction with breasts and 3 for physical well-being were described [32].

### Statistical analysis

Frequencies and proportions, means with range or standard deviation (SD), or medians with range or interquartile range (IQR), as appropriate, were used to describe baseline patient and treatment characteristics, as well as outcomes of the questionnaires. Additionally, these measures were employed to compare responders versus non-responders to questionnaires. The primary outcome included the comparison of long-term mean BREAST-Q and EORTC-QLQ-C30/BR23 scores by using an independent samples T-test. MCID values, as defined by previously published studies, were used to evaluate clinical relevance. [32, 33] All analyses were performed in IBM SPSS Statistics (26.0.0.1).

## Results

**Fig. 1:**
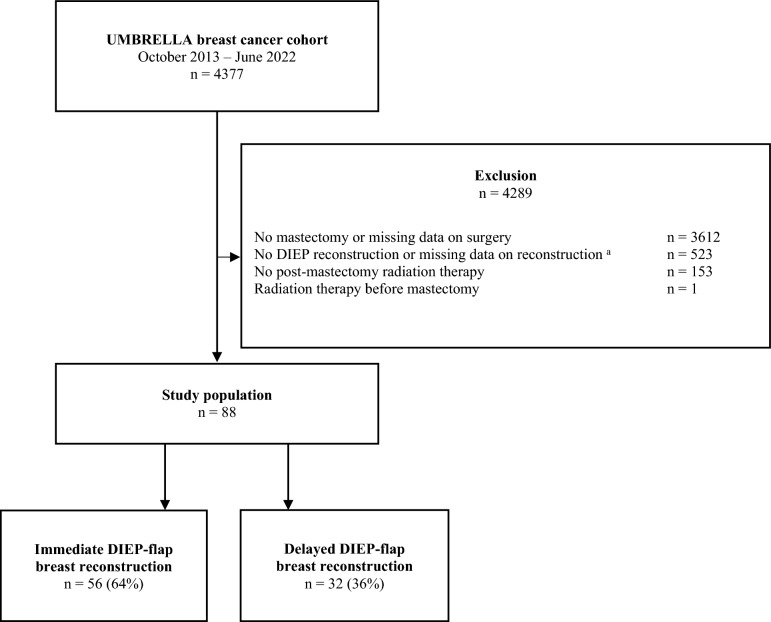
Flowchart of patient selection. **a** The Netherlands Cancer Registry does not document secondary breast reconstructions. In cases where patients were referred for their reconstruction to a hospital not affiliated with UMBRELLA, data regarding reconstruction is missing

Of the 88 UMBRELLA participants identified as eligible for this study, IBR was performed in 56 patients (64%) and 32 patients (36%) underwent DBR (Fig. 1). Patients had a mean age of 48.6 versus 47.0 years, respectively (Table 1). BMI in patients with DBR was higher (27.6 vs. 25.7 m/kg^2^). There were more former smokers in the DBR than in the IBR group (85.7% versus 48.4%) and there was only one current smoker among participants (IBR group). T-stage was similarly distributed between the two groups and the proportion of N-stage 2–3 was higher in the DBR group (20.6% versus 7.1%). A larger proportion of patients in the DBR group underwent axillary lymph node dissection (ALND; 21.6% vs. 10.7%). In both groups, most patients received locoregional radiotherapy without boost (75.0% and 71.9%, resp.). Median time interval between mastectomy and DBR was 15 months (range 7–62).

**Table 1:** Patient, tumor, and treatment characteristics of breast cancer patients treated with immediate versus delayed DIEP-flap breast reconstruction

	Immediate DIEP reconstruction		Delayed DIEP reconstruction	
Patient characteristics				
**Patients**, No. (%)	56	63.6	32	36.4
**Age**, mean (SD)	48.6	7.9	47.0	6.3
**Body Mass Index** ^a^, mean (SD)	25.7	3.1	27.6	3.6
Missing, No. (%)	25	44.6	18	56.3
**Smoking**, No. (%)				
Yes	1	3.2	0	0
No, never	15	48.4	2	14.3
No, stopped	15	48.4	12	85.7
Missing, No. (%)	25	44.6	18	56.3

Tumor characteristics ^b^				
**T-stage**, No. (%)				
0 + In situ	10	17.9	3	9.4
1	21	37.5	10	31.3
2	14	25.0	9	28.1
3 + 4	10	17.9	9	28.1
X + Unknown	1	1.8	1	3.1
**N-stage**, No. (%)				
0	11	19.6	10	29.4
1	37	66.1	15	47.1
2 + 3	4	7.1	5	20.6
X	4	7.1	0	0.0
**TNM-stage**, No %				
1	3	5.4	1	3.1
2	23	41.1	11	34.3
3	29	51.8	18	56.3
4	1	1.8	2	6.3

Treatment characteristics				
**Neo-adjuvant systemic therapy**, No. (%)				
Chemotherapy	36	64.3	19	59.4
Endocrine therapy	12	21.4	0	0.0
Immunotherapy	7	12.5	4	12.5
**Adjuvant systemic therapy**, No. (%)				
Chemotherapy	20	35.7	15	46.9
Endocrine therapy	46	82.1	25	78.1
Immunotherapy	10	17.9	6	18.8
**Axillary treatment**, No. (%)				
No axillary treatment	0	0.0	2	6.3
Sentinel node and/or MARI	50	89.3	23	71.9
ALND	6	10.7	7	21.9
**Radiotherapy** ^c^, No. (%)				
Local radiotherapy without boost	7	12.5	7	21.9
Local radiotherapy with boost	0	0.0	1	3.1
Locoregional radiotherapy without boost	42	75.0	23	71.9
Locoregional radiotherapy with boost	1	1.8	0	0.0
Other (e.g. regional lymph nodes only)	6	10.7	1	3.1
**Time between mastectomy and DIEP reconstruction in months**				
Median, range	0	0.0	15	7—62

*ALND* Axillary lymph node dissection; *DIEP* Deep Inferior Epigastric artery Perforator; *IQR* Inter quartile range; *MARI* Marking the axillary lymph node with radioactive iodine seeds; *SD* Standard deviation

^a^ Calculated as weight/height^2^

^b^ All tumor characteristics are classified according to the American Joint Committee for Cancer (AJCC) 7th/8th edition and refer to the pathological stages

^c^ Including radiation therapy on periclavicular and/or axillary lymph nodes

Baseline characteristics of responders versus non-responders to (any of the) questionnaires were presented in Table S1.

### Long-term patient-reported outcomes

At long-term follow-up (i.e., a minimum of 12 months post-reconstruction follow-up), the EORTC-QLQ-C30/BR23 was completed by 35 IBR patients and 16 DBR patients. Participants completed the EORTC-QLQ-C30/BR23 questionnaire at a median of 41 (range 13–85; IBR) and 62.5 (range 43–85; DBR) months after mastectomy (response rates: 63% and 50%, resp., Table S2). One patient in the IBR group was found to have been diagnosed with leukemia during follow-up and was therefore excluded from analysis of long-term PROs. Mean scores were comparable for both groups regarding body image (80.2 after IBR vs. 81.3 after DBR) and breast symptoms (16.9 vs. 16.7, resp., Table 2). When compared to IBR, patients reported slightly better outcomes regarding physical functioning (87.2 vs. 92.1, resp.) and pain (23.2 vs. 17.7, resp.) after DBR.

**Table 2:** Short and long-term patient-reported outcomes (PROs) of breast cancer patients who underwent immediate versus delayed Deep Inferior Epigastric artery Perforator (DIEP)-flap breast reconstruction using EORTC-QLQ-C30/BR23 and BREAST-Q questionnaire

EORTC-QLQ-C30/BR23 ^a^		6 months			12 months			Long term ^b^			
		*n*	Mean	SD	*n*	Mean	SD	*n*	Mean	SD	*p*-value
**Body image**	*IBR*	34	71.6	25.0	33	76.3	26.0	35	80.2	23.4	0.88
*DBR post-mastectomy*		20	62.9	24.1	12	66.7	28.9	16	81.3	19.4	
*DBR post-reconstruction*		17	79.9	17.9	14	86.9	11.7				
**Breast symptoms**	*IBR*	34	26.5	18.9	33	18.2	18.1	35	16.9	13.6	0.95
*DBR post-mastectomy*		21	28.2	18.2	12	20.1	14.4	16	16.7	13.6	
*DBR post-reconstruction*		17	17.1	18.3	14	17.3	22.3				
**Physical functioning**	*IBR*	34	86.5	12.7	33	87.3	14.1	35	87.2	14.2	0.22
*DBR post-mastectomy*		21	79.4	23.4	12	89.4	10.8	16	92.1	9.5	
*DBR post-reconstruction*		17	87.5	14.3	14	90.5	12.7				
**Pain**	*IBR*	34	24.5	20.2	33	19.7	23.0	35	23.2	23.2	0.37
*DBR post-mastectomy*		21	27.8	32.3	12	16.7	24.6	16	17.7	19.7	
*DBR post-reconstruction*		17	20.6	26.7	14	16.7	19.6				

			Long term ^d^			
BREAST-Q ^c^			*n*	Mean	SD	*p*-value
**Satisfaction with breasts**	*IBR*	N.A.^e^	26	66.9	14.3	0.85
	*DBR*		15	68.3	24.4	
**Physical well-being chest**	*IBR*	N.A.^e^	26	71.4	19.6	0.89
	*DBR*		14	70.5	20.6	
**Adverse effects of radiation**	*IBR*	N.A.^e^	12	89.0	14.0	0.77
	*DBR*		8	87.1	14.3	

*DBR* Delayed breast reconstruction; *DIEP* Deep Inferior Epigastric Artery Perforator; *IBR* Immediate breast reconstruction; *N.A.* Not applicable; *SD* Standard deviation

^a^ EORTC QLQ-C30 and -BR23 scores range from 0 to 100. Higher scores for body image and physical functioning represent better outcomes, whereas higher scores for breast symptoms and pain indicate a higher level of symptoms

^b^ Long-term outcomes were determined by using the last completed questionnaire with a minimum of 12 months after BR. Median long-term follow-up was 41 months for IBR and 46 months for DBR

^c^ BREAST-Q scores range from 0 to 100. Higher scores indicate better outcomes

^d^ Long-term outcomes were determined by using the last completed questionnaire with a minimum of 12 months after BR. Median long-term follow-up was 37 months for IBR and 42 months for DBR

^e^ The BREAST-Q is included to UMBRELLA questionnaires since August 2018, which means that data for 6 and 12 months follow-up is not available

The BREAST-Q questionnaire was completed by 26 IBR patients and 15 DBR patients at a median of 37 (range 13–90) and 73 (range 37–91) months after mastectomy, respectively (response rates: 46% and 47%, resp.). Patients reported similar satisfaction with breasts (66.9 after IBR vs. 68.3 after DBR), physical well-being chest (71.4 vs. 70.5, resp.), and adverse effects of radiation (89.0 vs. 87.1, resp., Table 2).

**Fig. 2:**
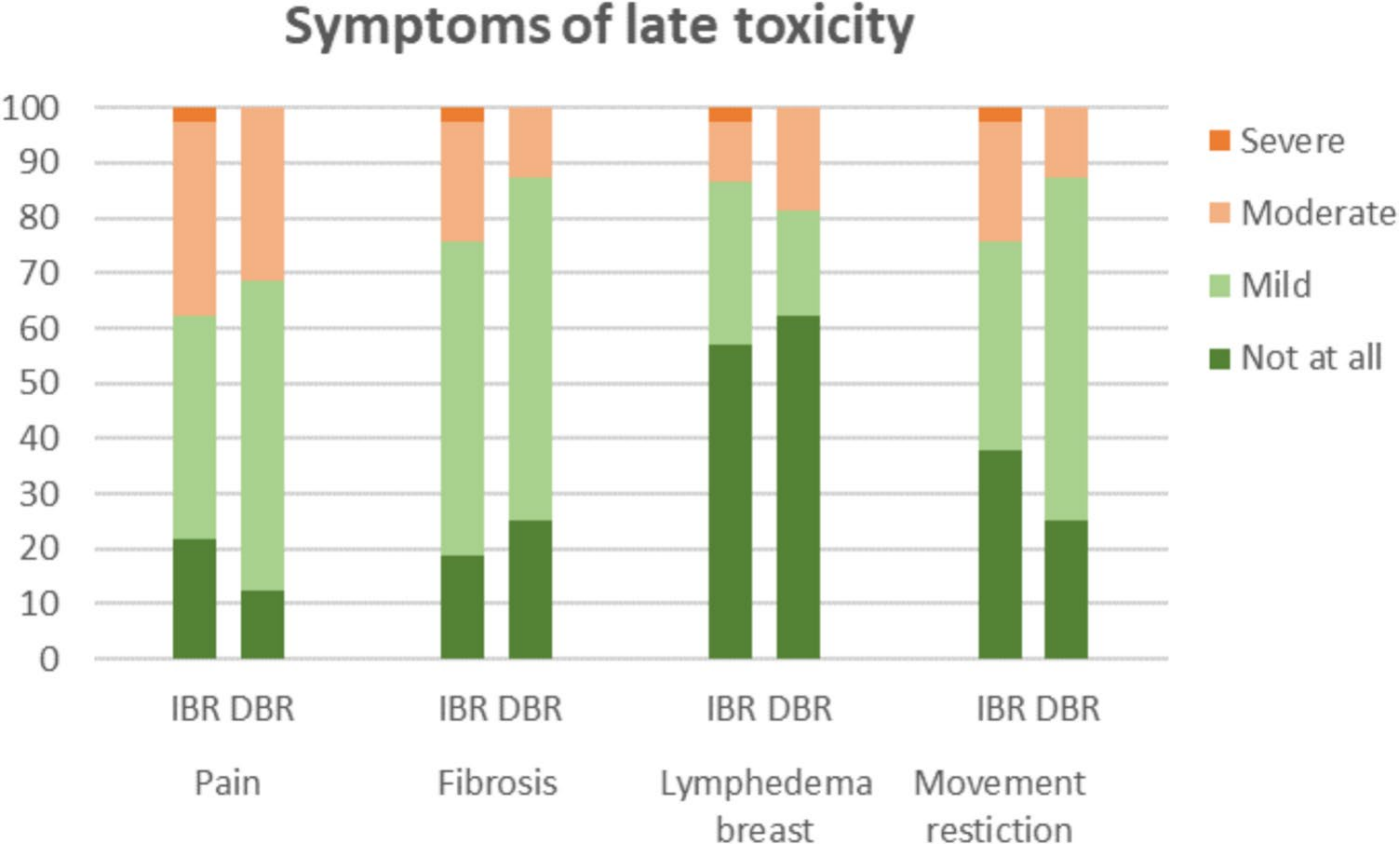
Long-term patient-reported late toxicity after a minimum of 12 months post-reconstruction. *IBR* Immediate Breast Reconstruction, *DBR* Delayed Breast Reconstruction

The LTT questionnaire was completed by 37 patients with IBR and 16 patients with DBR at a median of 29 (range 11–84) and 61 (range 28–83) months after mastectomy, respectively (response rates: 66% and 50%, resp.). Patients reported no large differences in moderate to severe pain (14/37; 37.8% vs. 5/16; 31.3%, resp.) and breast lymphedema (5/37; 13.5% vs. 3/16; 18.8%, resp., Fig. 2). A larger proportion of patients after IBR reported fibrosis (9/37; 24.3% vs. 2/16; 12.5%, resp.) and movement restriction (9/37; 24.3% vs. 2/16; 12.5%, resp.) compared to patients after DBR (Fig. 2).

### Patient-reported outcomes 6–12 months after mastectomy

Response rates ranged from 59–61% (IBR) to 53–63% (DBR) at the different time points (Table S1). Six months post-mastectomy, mean scores for body image (71.6 after IBR vs. 62.9 after DBR), breast symptoms (26.5 vs. 28.2, resp.), physical functioning (86.5 vs. 79.4, resp.), and pain (24.5 vs. 27.8, resp.) were more favorable for patients with IBR compared to patients after DBR (Table 2).

Twelve months post-mastectomy, mean scores for body image (76.3 after IBR vs. 69.6 after DBR), breast symptoms (18.2 vs. 23.5, resp.), and physical functioning (87.3 vs. 86.3, resp.) continued to be more favorable for IBR, while mean scores for pain were lower in the DBR group (19.7 vs. 16.7, resp.).

### Patient-reported outcomes 6–12 months after reconstruction

Response rates ranged from 59–61% (IBR) and 44–53% (DBR) for the different time points (Table S2). Six months post-reconstruction, mean scores for body image (71.6 vs. 79.9), breast symptoms (17.1 vs. 26.5), and pain (24.5 vs. 20.6) were more favorable for patients with DBR compared to patients with IBR (Table 2).

Twelve months post-reconstruction, mean scores for body image (76.3 vs. 86.9), physical functioning (87.3 vs. 90.5), and pain (19.7 vs. 16.7) continued to be more favorable for patients with DBR.

## Discussion

In this study, both short and long-term patient-reported outcomes were compared between patients who underwent immediate breast reconstruction versus delayed breast reconstruction and received post-mastectomy radiotherapy. Also, PROs in the trajectory after mastectomy and reconstruction for both groups were measured. In the initial year following mastectomy, patients with IBR showed more favorable scores compared to patients with DBR, especially for body image and physical functioning. Conversely, in the first year after the reconstruction, patients with DBR had slightly better PROs compared to those with IBR. In the long term, patients who underwent IBR + PMRT reported more fibrosis and movement restriction, but no statistically significant differences were observed in patient-reported satisfaction and HR-QoL.

Taking into account the aforementioned MCID values, the only clinically relevant difference was observed in physical functioning (EORTC-QLQ-C30) at 6 months, favoring IBR. No other differences were observed from 12 months onwards. Although no MCID has been established yet for body image, it is, however, likely that the observed differences for body image at 6 and 12 months post-mastectomy (MD = 8.7, and MD = 9.6, resp.) are also clinically relevant, favoring IBR. [34] This suggests that patients undergoing IBR experience immediate benefits from their reconstruction in the initial year, whereas DBR patients must live through a period without their breast(s). As expected, following the completion of DBR, PROs improved to comparative levels as the IBR group. Additional benefits of IBR include the avoidance of a second surgery, allowing patients to recover from only one procedure instead of two. Furthermore, IBR offers the opportunity to preserve the skin envelope and nipple-areola complex.

Following the entire timeline, it becomes evident that patients with DBR experience a period of reduced body image and poorer physical functioning shortly after mastectomy. Although patients who underwent IBR had more extensive primary surgery, we did not observe more pain and even observed better scores for physical functioning in this group at 6 months following mastectomy. This could be explained by the psychological impact of the loss of their breast(s) in DBR patients, even affecting these physical domains. Another explanation could be that more patients underwent ALND in this group and this axillary surgery affected pain and physical functioning scores.

Existing literature suggests that patients who experience temporary breast loss tend to report higher satisfaction after finally receiving breast reconstruction, which is reflected in post-reconstruction outcomes. [35] This phenomenon may be attributed to the comparison of their reconstructed breast to their previous flat chest state, which potentially explains the notable differences in post-reconstruction PROs favoring DBR. In contrast, patients who undergo IBR tend to compare their reconstructed breast to their preoperative untouched breast. [36, 37] Furthermore, the difference in oncological timelines of the two groups may impact short-term post-reconstruction outcomes. For example, patients with IBR might not have completed their oncological treatment (e.g., adjuvant systemic therapy) yet. Nonetheless, long-term PROs become comparable between the two groups. Other studies that compared PROs for IBR versus DBR in the setting of PMRT [18, 36, 38, 39] found similar results.

A substantial proportion of patients undergoing radiotherapy for breast cancer report symptoms of late toxicity. [30] In our study, patients who underwent IBR more frequently reported symptoms of fibrosis and movement restriction in comparison to patients who underwent DBR (24.3% versus 12.5%), at a median follow-up of 29 and 60 months post-mastectomy, respectively. However, long-term PROs as measured by EORTC-QLQ-C30/BR23 and BREAST-Q showed comparable results on all subdomains. This finding suggests that while IBR may result in more symptoms of fibrosis and movement restriction, patients may not experience this as invalidating, as it does not affect patients’ self-reported long-term HR-QoL and satisfaction. A systematic review by Kelley et al. reported a pooled rate of 27% for flap fibrosis in autologous reconstructions exposed to radiotherapy, which aligns closely with our results. [40] However, a more clinically relevant question would be how the severity of the fibrosis impacts patients’ satisfaction and QoL. For instance, neobreasts requiring revision surgery due to severe fibrosis would likely be considered more detrimental than a slightly firmer or less ptotic breast. Mirzabegi et al., who also found comparable rates of fibroses of patient who received PMRT after IBR, reported similar rates of revision surgery when compared to IBR without PMRT at a follow-up of 18.7 months. [21] However, long-term studies are needed as the effects of radiotherapy may become visible up to years after treatment.

Due to the low number of patients with immediate-delayed reconstruction (*n* = 3) in our cohort, we chose to exclude them from analysis. Consequently, all patients in the DBR group in our study underwent a simple mastectomy followed by a delayed DIEP-flap BR. In this approach, abdominal skin replaces the breast skin in the lower pole, thus lacking the advantage of preserving the breast skin envelope and possibly the nipple. Although preservation of the breast skin envelope is one of the benefits of immediate-delayed BR, this approach is associated with significantly different clinical and psychological outcomes. [41, 42] A recent development involves the introduction of preoperative radiotherapy followed by mastectomy and IBR. [43, 44] This approach may be an alternative worth considering in the future, but adequately powered high-quality studies need to be completed first.

Although changes in the NCCN guidelines have led to increased application of PMRT, its use in patients with one to three positive lymph nodes remains a topic of debate. [13, 45] In our study, patients in the DBR group had larger tumors and more lymph nodes were involved. One possible explanation is that, at the time, the decision for delayed reconstruction was made in anticipation of potential PMRT. Studies investigating factors that predict the need for PMRT would provide significant added value. In line with this, Chen et al. recently developed a machine learning model to predict the need for PMRT after IBR in a clinical setting [46].

This study has some limitations. First, despite being a multicenter study, the fact that nearly all IBRs were performed at a single center, combined with a low number of eligible participants, made it challenging to draw definitive conclusions. Moreover, the study population was too small to adjust for potential relevant confounders (i.e., age, educational level, BMI, smoking, systemic therapy, axillary treatment, and radiotherapy). This limitation is most likely due to the rareness of PMRT after direct autologous reconstructions in the Netherlands, but also worldwide. Given that only a few centers in the Netherlands offer this particular approach, it is likely that we included almost all patients (willing to participate in research) with irradiated IBRs in the Netherlands. Second, we only assessed PROs, which although invaluable and clinically relevant, may not be directly related to other outcomes such as objective measures of fibrosis, atrophy or clinicians’ opinions. [36, 38] Last, no pre-operative PRO data were available from UMBRELLA participants. A strength of this study is that it is unique in its longitudinal and long-term follow-up of patients receiving PMRT combined with immediate or delayed DIEP-flap reconstruction, and in utilizing PRO questionnaires to assess results. It is one of few prospective studies with a longitudinal collection of PROs, following patients over time in their journey of breast cancer treatment and breast reconstruction. [18] Extended follow-up periods involving larger cohorts will be necessary to gain further insights into the effects of PMRT on IBR and to formulate definite conclusions. Through this research, we encourage other researchers to undertake studies on this timely topic.

## Conclusion

In this prospective study, long-term PROs were comparable between immediate and delayed DIEP-flap breast reconstruction with PMRT, and therefore, both treatment pathways should be considered in routine care taking patients’ preferences into account.

In the initial year following mastectomy, patients with DBR experience a period of reduced body image and poorer physical functioning while awaiting their final breast reconstruction. In the time after their DBR, their scores demonstrated improvement. Conversely, patients with IBR experience immediate benefits from the reconstruction, but exhibit a higher incidence of long-term radiotherapy-related toxicity without impacting satisfaction or HR-QoL. Overall, there are no substantial differences that render one method superior and the choice should be based on patients’ individual needs and preferences.

These findings can be used to counsel patients and manage expectations for those aiming to undergo an autologous breast reconstruction and PMRT. This will support patients and clinicians in reaching a well-informed, personalized shared decision.

## Supplementary Information

Below is the link to the electronic supplementary material.

Supplementary file1 (DOCX 348 KB)

## Data Availability

The datasets analyzed during the current study are available from the corresponding author on reasonable request.

## Abbreviations

*BCS*: Breast Conserving Surgery
*BR*: Breast Reconstruction
*DCIS*: Ductal Carcinoma In Situ
*DBR*: Delayed Breast Reconstruction
*DIEP*: Deep Inferior Epigastric Artery Perforator
*EORTC*: European Organization for Research and Treatment
*IBR*: Immediate Breast Reconstruction
*MCID*: Minimal Clinically Relevant Difference
*MREC*: Medical Research Ethics Committee
*NCR*: Netherlands Cancer Registry
*NCCN*: Netherlands Comprehensive Cancer Network
*PMRT*: Post-Mastectomy Radiotherapy
*PROs*: Patient-Reported Outcomes
*HR-QoL*: Health-Related Quality of Life
*UMBRELLA*: Utrecht Cohort for Multiple BREast Cancer Intervention Studies and Long-Term Evaluation

## References

[1] Greenberg CC, Lipsitz SR, Hughes ME et al (2011) Institutional variation in the surgical treatment of breast cancer: a study of the NCCN. Ann Surg 254(2):339–345. 10.1097/SLA.0b013e3182263bb0 21725233 10.1097/SLA.0b013e3182263bb0 PMC3428030

[2] Zehra S, Doyle F, Barry M, Walsh S, Kell MR (2020) Health-related quality of life following breast reconstruction compared to total mastectomy and breast-conserving surgery among breast cancer survivors: a systematic review and meta-analysis. Breast Cancer 27(4):534–566. 10.1007/s12282-020-01076-1 32162181 10.1007/s12282-020-01076-1

[3] Jagsi R, Momoh AO, Qi J et al (2018) Impact of radiotherapy on complications and patient-reported outcomes after breast reconstruction. J Natl Cancer Inst 110(2):157–165. 10.1093/jnci/djx148 28954300 10.1093/jnci/djx148 PMC6059091

[4] Myers PL, Nelson JA, Allen RJ Jr (2021) Alternative flaps in autologous breast reconstruction. Gland Surg 10(1):444–459 33634002 10.21037/gs.2020.03.16 PMC7882326

[5] Razdan SN, Cordeiro PG, Albornoz CR et al (2017) National breast reconstruction utilization in the setting of postmastectomy radiotherapy. J Reconstr Microsurg 33(5):312–317. 10.1055/s-0037-1598201 28235218 10.1055/s-0037-1598201 PMC5885287

[6] Schaverien MV, Macmillan RD, McCulley SJ (2013) Is immediate autologous breast reconstruction with postoperative radiotherapy good practice?: a systematic review of the literature. J Plast Reconstr Aesthet Surg 66(12):1637–1651. 10.1016/j.bjps.2013.06.059 23886555 10.1016/j.bjps.2013.06.059

[7] Khajuria A, Farhadi J (2020) Immediate versus delayed autologous breast reconstruction. J Plast Reconstr Aesthet Surg 73(5):983–1007. 10.1016/j.bjps.2019.12.013 32005638 10.1016/j.bjps.2019.12.013

[8] Zhong T, Hu J, Bagher S et al (2016) A comparison of psychological response, body image, sexuality, and quality of life between immediate and delayed autologous tissue breast reconstruction: a prospective long-term outcome study. Plast Reconstr Surg 138(4):772–780. 10.1097/prs.0000000000002536 27673514 10.1097/PRS.0000000000002536

[9] Heimes AS, Stewen K, Hasenburg A (2017) Psychosocial aspects of immediate versus delayed breast reconstruction. Breast Care (Basel) 12(6):374–377. 10.1159/000485234 29456468 10.1159/000485234 PMC5803679

[10] van Bommel ACM, de Ligt KM, Schreuder K et al (2020) The added value of immediate breast reconstruction to health-related quality of life of breast cancer patients. Eur J Surg Oncol. 10.1016/j.ejso.2020.06.009 32763107 10.1016/j.ejso.2020.06.009

[11] Chao LF, Patel KM, Chen SC et al (2014) Monitoring patient-centered outcomes through the progression of breast reconstruction: a multicentered prospective longitudinal evaluation. Breast Cancer Res Treat 146(2):299–308. 10.1007/s10549-014-3022-7 24951266 10.1007/s10549-014-3022-7

[12] Ochoa O, Garza R 3rd, Pisano S et al (2022) Prospective longitudinal patient-reported satisfaction and health-related quality of life following DIEP flap breast reconstruction: effects of reconstruction timing. Plast Reconstr Surg 149(5):848e–857e. 10.1097/prs.0000000000009044 35245253 10.1097/PRS.0000000000009044

[13] McGale P, Taylor C, Correa C et al (2014) Effect of radiotherapy after mastectomy and axillary surgery on 10-year recurrence and 20-year breast cancer mortality: meta-analysis of individual patient data for 8135 women in 22 randomised trials. Lancet 383(9935):2127–2135. 10.1016/s0140-6736(14)60488-8 24656685 10.1016/S0140-6736(14)60488-8 PMC5015598

[14] Frasier LL, Holden S, Holden T et al (2016) Temporal trends in postmastectomy radiation therapy and breast reconstruction associated with changes in national comprehensive cancer network guidelines. JAMA Oncol 2(1):95–101. 10.1001/jamaoncol.2015.3717 26539936 10.1001/jamaoncol.2015.3717 PMC4713236

[15] Crisera CA, Chang EI, Da Lio AL, Festekjian JH, Mehrara BJ (2011) Immediate free flap reconstruction for advanced-stage breast cancer: is it safe? Plast Reconstr Surg 128(1):32–41. 10.1097/PRS.0b013e3182174119 21399562 10.1097/PRS.0b013e3182174119

[16] Tran NV, Chang DW, Gupta A, Kroll SS, Robb GL (2001) Comparison of immediate and delayed free TRAM flap breast reconstruction in patients receiving postmastectomy radiation therapy. Plast Reconstr Surg 108(1):78–82. 10.1097/00006534-200107000-00013 11420508 10.1097/00006534-200107000-00013

[17] Kronowitz SJ, Robb GL (2004) Breast reconstruction with postmastectomy radiation therapy: current issues. Plast Reconstr Surg 114(4):950–960. 10.1097/01.prs.0000133200.99826.7f 15468404 10.1097/01.prs.0000133200.99826.7f

[18] Billig J, Jagsi R, Qi J et al (2017) Should immediate autologous breast reconstruction be considered in women who require postmastectomy radiation therapy? a prospective analysis of outcomes. Plast Reconstr Surg 139(6):1279–1288. 10.1097/prs.0000000000003331 28198770 10.1097/PRS.0000000000003331 PMC5676536

[19] Jassem J (2017) Post-mastectomy radiation therapy after breast reconstruction: Indications, timing and results. Breast 34(Suppl 1):S95-s98. 10.1016/j.breast.2017.06.037 28673536 10.1016/j.breast.2017.06.037

[20] Taghizadeh R, Moustaki M, Harris S, Roblin P, Farhadi J (2015) Does post-mastectomy radiotherapy affect the outcome and prevalence of complications in immediate DIEP breast reconstruction? a prospective cohort study. J Plast Reconstr Aesthet Surg 68(10):1379–1385. 10.1016/j.bjps.2015.06.003 26210234 10.1016/j.bjps.2015.06.003

[21] Mirzabeigi MN, Smartt JM, Nelson JA, Fosnot J, Serletti JM, Wu LC (2013) An assessment of the risks and benefits of immediate autologous breast reconstruction in patients undergoing postmastectomy radiation therapy. Ann Plast Surg 71(2):149–155. 10.1097/SAP.0b013e31824b3dcc 23542828 10.1097/SAP.0b013e31824b3dcc

[22] Schreuder K, van Bommel ACM, de Ligt KM et al (2017) Hospital organizational factors affect the use of immediate breast reconstruction after mastectomy for breast cancer in the Netherlands. Breast 34:96–102. 10.1016/j.breast.2017.05.011 28552797 10.1016/j.breast.2017.05.011

[23] van Bommel AC, Mureau MA, Schreuder K et al (2017) Large variation between hospitals in immediate breast reconstruction rates after mastectomy for breast cancer in the Netherlands. J Plast Reconstr Aesthet Surg 70(2):215–221. 10.1016/j.bjps.2016.10.022 27993547 10.1016/j.bjps.2016.10.022

[24] van Bommel AC, Spronk PE, Vrancken Peeters MT et al (2017) Clinical auditing as an instrument for quality improvement in breast cancer care in the Netherlands: the national NABON Breast Cancer Audit. J Surg Oncol 115(3):243–249. 10.1002/jso.24516 27885679 10.1002/jso.24516

[25] Young-Afat DA, van Gils CH, van den Bongard HJGD et al (2017) The Utrecht cohort for Multiple BREast cancer intervention studies and long-term evaLuAtion (UMBRELLA): objectives, design, and baseline results. Breast Cancer Res Treat 164(2):445–450. 10.1007/s10549-017-4242-4 28444532 10.1007/s10549-017-4242-4 PMC5487711

[26] Sawaki M, Shien T, Iwata H (2019) TNM classification of malignant tumors (Breast Cancer Study Group). Jpn J Clin Oncol 49(3):228–231. 10.1093/jjco/hyy182 30541035 10.1093/jjco/hyy182

[27] Aaronson NK, Ahmedzai S, Bergman B et al (1993) The European organization for research and treatment of cancer QLQ-C30: a quality-of-life instrument for use in international clinical trials in oncology. J Natl Cancer Inst 85(5):365–376. 10.1093/jnci/85.5.365 8433390 10.1093/jnci/85.5.365

[28] Fayers PM AN, Bjordal K, Groenvold M, Curran D, Bottomley A, on behalf of the EORTC Quality of Life Group. The EORTC QLQ-C30 Scoring Manual (3rd Edition). *European Organisation for Research and Treatment of Cancer, Brussels 2001.* 2001.

[29] Pusic AL, Klassen AF, Scott AM, Klok JA, Cordeiro PG, Cano SJ (2009) Development of a new patient-reported outcome measure for breast surgery: the BREAST-Q. Plast Reconstr Surg 124(2):345–353. 10.1097/PRS.0b013e3181aee807 19644246 10.1097/PRS.0b013e3181aee807

[30] Batenburg MCT, Mink van der Molen DR, van der Leij F et al (2023) Patient-reported symptoms of late toxicity in patients with breast cancer treated with hypofractionated radiation therapy and the association with quality of life. Int J Radiat Oncol Biol Phys 115(5):1181–1191. 10.1016/j.ijrobp.2022.11.008 36402357 10.1016/j.ijrobp.2022.11.008

[31] Jaeschke R, Singer J, Guyatt GH (1989) Measurement of health status. ascertaining the minimal clinically important difference. Control Clin Trials 10(4):407–415 2691207 10.1016/0197-2456(89)90005-6

[32] Voineskos SH, Klassen AF, Cano SJ, Pusic AL, Gibbons CJ (2020) Giving meaning to differences in BREAST-Q scores: minimal important difference for breast reconstruction patients. Plast Reconstr Surg 145(1):11e–20e. 10.1097/prs.0000000000006317 31577663 10.1097/PRS.0000000000006317

[33] Musoro JZ, Coens C, Fiteni F et al (2019) Minimally important differences for interpreting EORTC QLQ-C30 scores in patients with advanced breast cancer. JNCI Cancer Spectr. 10.1093/jncics/pkz037 32328553 10.1093/jncics/pkz037 PMC7050000

[34] Sprangers MA, Groenvold M, Arraras JI et al (1996) The European organization for research and treatment of cancer breast cancer-specific quality-of-life questionnaire module: first results from a three-country field study. J Clin Oncol 14(10):2756–2768. 10.1200/jco.1996.14.10.2756 8874337 10.1200/JCO.1996.14.10.2756

[35] Brorson F, Elander A, Thorarinsson A, Hansson E (2022) Patient reported outcome and quality of life after delayed breast reconstruction—an RCT comparing different reconstructive methods in radiated and non-radiated patients. Clin Breast Cancer 22(8):753–761. 10.1016/j.clbc.2022.09.004 36210311 10.1016/j.clbc.2022.09.004

[36] Steele KH, Macmillan RD, Ball GR, Akerlund M, McCulley SJ (2018) Multicentre study of patient-reported and clinical outcomes following immediate and delayed autologous breast reconstruction and radiotherapy (ABRAR study). J Plast Reconstr Aesthet Surg 71(2):185–193. 10.1016/j.bjps.2017.10.030 29203259 10.1016/j.bjps.2017.10.030

[37] O’Connell RL, Di Micco R, Khabra K et al (2018) Comparison of immediate versus delayed DIEP flap reconstruction in women who require postmastectomy radiotherapy. Plast Reconstr Surg 142(3):594–605. 10.1097/prs.0000000000004676 29927832 10.1097/PRS.0000000000004676 PMC6112844

[38] Adesiyun TA, Lee BT, Yueh JH et al (2011) Impact of sequencing of postmastectomy radiotherapy and breast reconstruction on timing and rate of complications and patient satisfaction. Int J Radiat Oncol Biol Phys 80(2):392–397. 10.1016/j.ijrobp.2010.02.039 20584583 10.1016/j.ijrobp.2010.02.039

[39] Lee BT et al (2010) Postmastectomy radiation therapy and breast reconstruction: an analysis of complications and patient satisfaction. Ann Plast Surg 64(5):679–683. 10.1097/SAP.0b013e3181db7585 20395800 10.1097/SAP.0b013e3181db7585

[40] Kelley BP, Ahmed R, Kidwell KM, Kozlow JH, Chung KC, Momoh AO (2014) A systematic review of morbidity associated with autologous breast reconstruction before and after exposure to radiotherapy: are current practices ideal? Ann Surg Oncol 21:1732–1738 24473643 10.1245/s10434-014-3494-z PMC4153351

[41] Shammas RL, Gordee A, Lee HJ et al (2023) Complications, costs, and healthcare resource utilization after staged, delayed, and immediate free-flap breast reconstruction: a longitudinal claims-based analysis. Ann Surg Oncol 30(4):2534–2549. 10.1245/s10434-022-12896-0 36474094 10.1245/s10434-022-12896-0 PMC9735033

[42] Shammas RL, Sergesketter AR, Taskindoust M et al (2021) An assessment of patient satisfaction and decisional regret in patients undergoing staged free-flap breast reconstruction. Ann Plast Sur. 10.1097/sap.0000000000002699 10.1097/SAP.0000000000002699 34100812

[43] Thiruchelvam PTR, Leff DR, Godden AR et al (2022) Primary radiotherapy and deep inferior epigastric perforator flap reconstruction for patients with breast cancer (PRADA): a multicentre, prospective, non-randomised, feasibility study. Lancet Oncol 23(5):682–690. 10.1016/S1470-2045(22)00145-0 35397804 10.1016/S1470-2045(22)00145-0 PMC9630150

[44] Godden AR, Micha A, O’Connell RL et al (2023) Pre-operative radiotherapy and deep inferior epigastric artery perforator (DIEP) flAp study (PRADA): aesthetic outcome and patient satisfaction at one year. J Plast Reconstr Aesthet Surg 78:19–28. 10.1016/j.bjps.2022.11.040 36764040 10.1016/j.bjps.2022.11.040

[45] Sekiguchi K (2017) Controversies in the role of postmastectomy radiotherapy in breast cancer patients with one to three positive axillary nodes and safety of integrating radiotherapy and breast reconstruction. Breast Cancer 24(4):493–495 28616687 10.1007/s12282-017-0788-6

[46] Chen YF, Chawla S, Mousa-Doust D, Nichol A, Ng R, Isaac KV (2024) Machine learning to predict the need for postmastectomy radiotherapy after immediate breast reconstruction. Plast Reconstr Surg Glob Open 12(2):e5599. 10.1097/gox.0000000000005599 38322813 10.1097/GOX.0000000000005599 PMC10846766

