# Supplementary material for: Patient-reported outcomes after immediate and delayed DIEP-flap breast reconstruction in the setting of post-mastectomy radiation therapy—results of the multicenter UMBRELLA breast cancer cohort
Source: Breast Cancer Res Treat. 2025 Feb 3;210(3):759–69. doi: 10.1007/s10549-025-07613-w (PMC11953195; doi:10.1007/s10549-025-07613-w)
Supplement: Supplementary file 1 — Supplementary file1 (DOCX 348 KB) [file 10549_2025_7613_MOESM1_ESM.docx]

**Supplementary information**

Appendices

**Appendix I**. The late treatment toxicity questionnaire

Supplementary tables

**Table 1**. Baseline characteristics of responders and non-responders to questionnaires

**Table 2**. Response rates to questionnaires

**Appendix I.** The late treatment toxicity questionnaire.


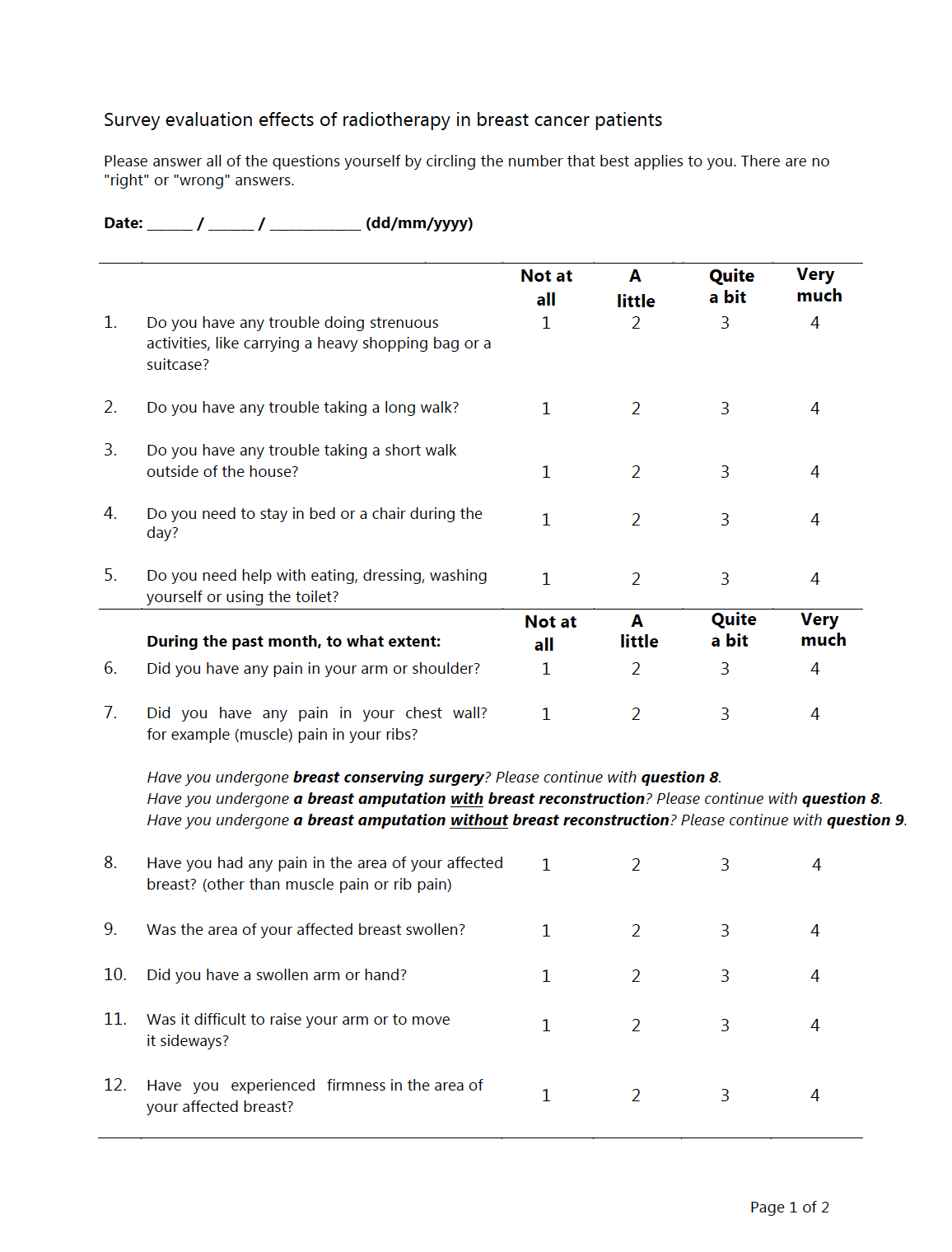

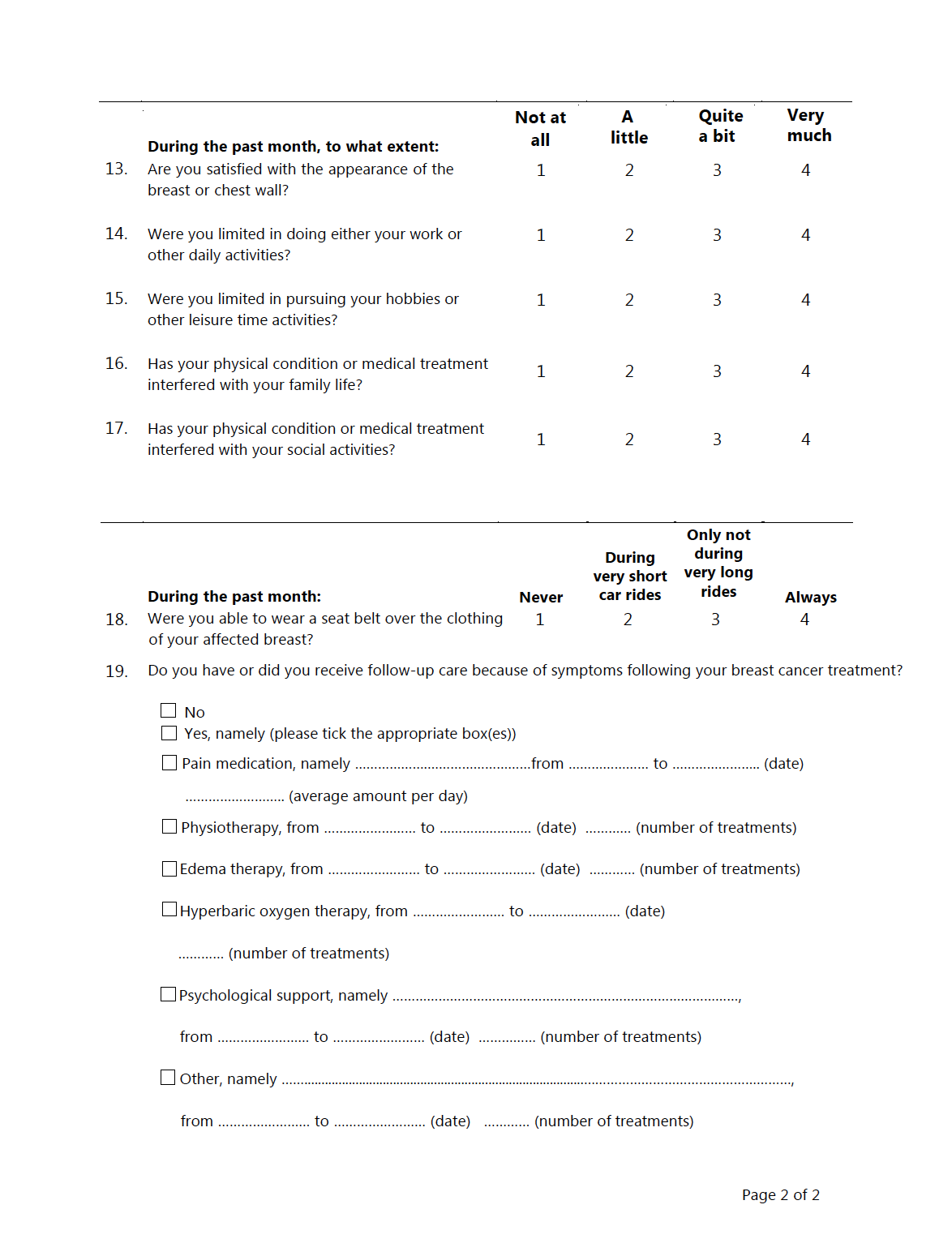


| **Supplementary Table 1**. Baseline characteristics of responders and non-responders to questionnaires. | | | | |
| --- | --- | --- | --- | --- |
|  | **Responders** | | **Non-responders** | |
|  | **n = 84 (95.5%)** | | **n=4 (4.5%)** | |
| **Patient characteristics ^a^** |  |  |  |  |
| Age, mean (SD) | 48.3 | (7.4) | 42.8 | (5.1) |
| **Tumor characteristics** |  |  |  |  |
| T-stadium, No. (%) |  |  |  |  |
| 0 + In situ | 11 | (13.1) | 2 | (50.0) |
| 1 | 30 | (35.7) | 1 | (25.0) |
| 3 | 22 | (26.2) | 1 | (25.0) |
| 3 + 4 | 19 | (22.6) | 0 | (0.0) |
| X + Unknown | 2 | (2.4) | 0 | (0.0) |
| N-stadium, No. (%) |  |  |  |  |
| 0 | 19 | (22.6) | 2 | (50.0) |
| 1 | 50 | (59.5) | 2 | (50.0) |
| 2 + 3 | 11 | (13.1) | 0 | (0.0) |
| X | 4 | (4.8) | 0 | (0.0) |
| **Treatment characteristics** |  |  |  |  |
| Neo-adjuvant systemic therapy, No. (%) |  |  |  |  |
| Chemotherapy | 52 | (61.9) | 3 | (75.0) |
| Endocrine therapy | 11 | (13.1) | 1 | (25.0) |
| Immunotherapy | 10 | (11.9) | 1 | (25.0) |
| Adjuvant systemic therapy, No. (%) |  |  |  |  |
| Chemotherapy | 45 | (40.5) | 1 | (25.0) |
| Endocrine therapy | 68 | (81.0) | 3 | (75.0) |
| Immunotherapy | 15 | (17.9) | 1 | (25.0) |
| Axillary treatment, No. (%) |  |  |  |  |
| No axillary treatment | 2 | (2.4) | 0 | (0.0) |
| Sentinel node and/or MARI | 69 | (82.1) | 4 | (100.0) |
| ALND | 13 | (15.5) | 0 | (0.0) |
| Radiotherapy^b^, No. (%) |  |  |  |  |
| Local radiotherapy without boost | 14 | (16.7) | 0 | (0.0) |
| Local radiotherapy with boost | 1 | (1.2) | 0 | (0.0) |
| Locoregional radiotherapy without boost | 59 | (70.2) | 3 | (75.0) |
| Locoregional radiotherapy with boost | 1 | (1.2) | 0 | (0.0) |
| Other (e.g. regional lymph nodes only) | 9 | (10.7) | 1 | (25.0) |
| Time between mastectomy and DIEP reconstruction in months |  |  |  |  |
| Median, range | 0 | (0-62) | 0 | (0-12) |
| *Abbreviations:* ALND = Axillary lymph node dissection; DIEP = Deep Inferior Epigastric artery Perforator; IQR = Inter quartile range; MARI = Marking the axillary lymph node with radioactive iodine seeds; SD = Standard deviation. | | | | |
| ^a^ Due to non-response to the questionnaires, data pertaining to BMI and smoking were unavailable. | | | | |
| ^b^ Including radiation therapy on periclavicular and/or axillary lymph nodes | | | |  |

**Supplementary Table 2**. Response rates to questionnaires.

|  | **IBR** | | **DBR** | | |  |
| --- | --- | --- | --- | --- | --- | --- |
|  | n | (%) | | n | (%) | |
| **EORTC-QLQ-C30/BR23** |  |  | |  |  | |
| 6 months post-mastectomy | 34 | (61) | | 20 | (63) | |
| 12 months post-mastectomy | 33 | (59) | | 17 | (53) | |
| 6 months post-reconstruction | 34 | (61) | | 17 | (53) | |
| 12 months post-reconstruction | 33 | (59) | | 14 | (44) | |
| Long term | 35 | (63) | | 16 | (50) | |
| **BREAST-Q** |  |  | |  |  | |
| Long term | 26 | (46) | | 15 | (47) | |
| **Late Treatment Toxicity** |  |  | |  |  | |
| Long term | 37 | (66) | | 16 | (50) | |
| *Abbreviations*: DBR = delayed breast reconstruction, IBR = immediate breast reconstruction | | | | | |  |
